# Supplementary material for: Multiple parameters shape the 3D chromatin structure of single nuclei at the doc locus in Drosophila
Source: Nat Commun. 2022 Sep 14;13:5375. doi: 10.1038/s41467-022-32973-y (PMC9474875; doi:10.1038/s41467-022-32973-y)
Supplement: Supplementary file 1 — Supplementary Information [file 41467_2022_32973_MOESM1_ESM.pdf]

SUPPLEMENTARY INFORMATION RELATED TO

Multiple parameters shape the 3D chromatin structure of single nuclei at the doc locus in *Drosophila*

Author list

Markus Götz[1,2], Olivier Messina[1], Sergio Espinola[1], Jean-Bernard Fiche[1], Marcelo Nollmann[1*]

Affiliations

[1] Centre de Biologie Structurale, Univ Montpellier, CNRS UMR 5048, INSERM U1054, Montpellier, France.

[2] current address: PicoQuant GmbH, Rudower Chaussee 29, 12489 Berlin, Germany

[*] corresponding author: marcelo.nollmann@cbs.cnrs.fr

Supplementary Figures

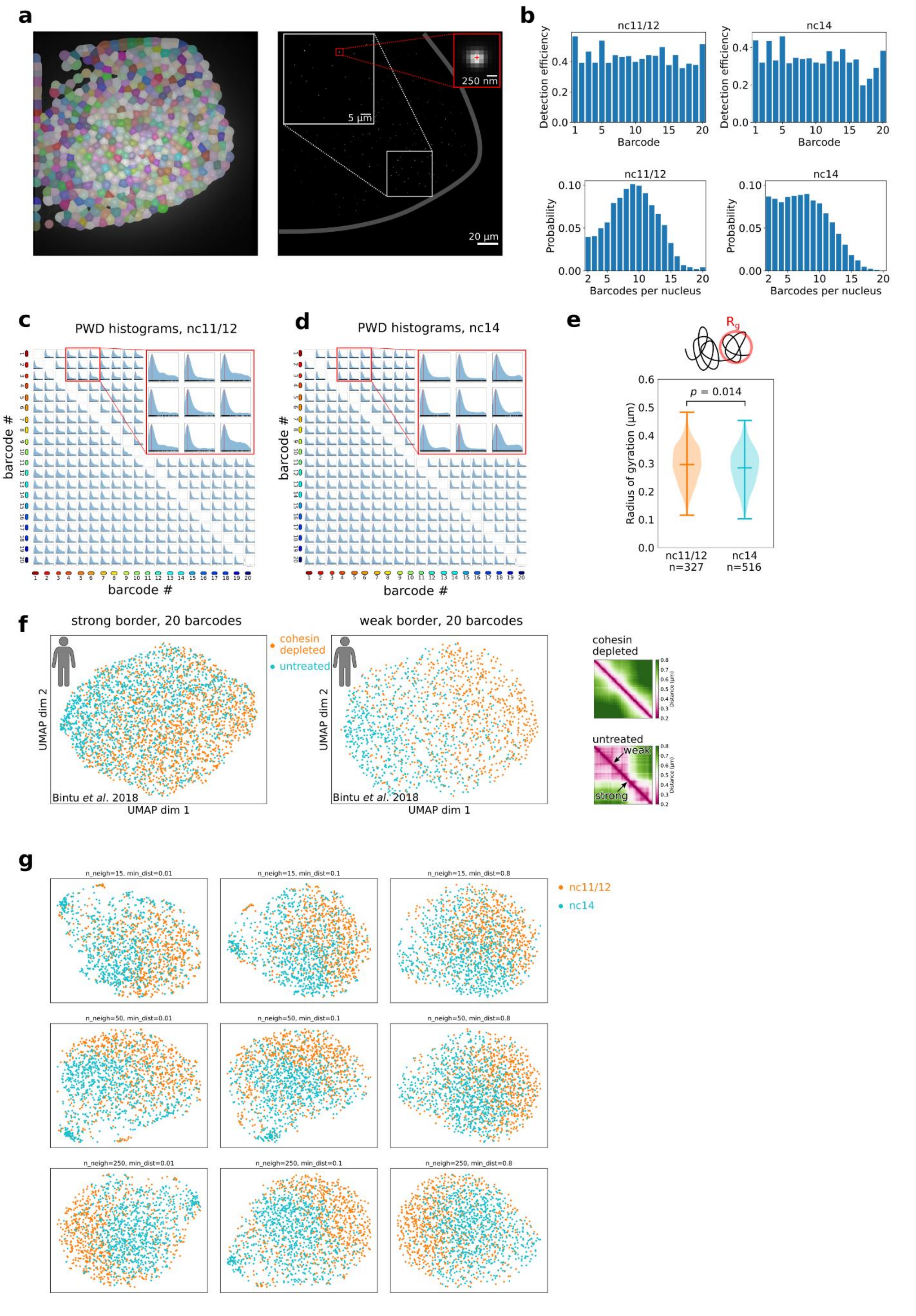

Supplementary Figure 1. HiM statistics and UMAP validation.

a. Left: Greyscale image of DAPI-stained nuclei from a *Drosophila* embryo overlaid with the extracted masks after nuclei segmentation. Right: Typical maximum intensity projection of the fluorescence signal from a single barcode in the same field of view as to the left. The

border of the embryo is shown by a thick gray line. The red-framed inset shows the point spread function of one barcode and indicates the localization of the center with a red cross.

b. Top: Detection efficiency for all barcodes, according to nuclear cycle. Bottom: Distribution of the number of detected barcodes per nucleus.

c. Map of pairwise distance distributions for all barcode combinations in nc11/12 nuclei. The order of the distributions follows that in the ensemble pairwise distance map (Fig. 1b). The blue shade represents a kernel density estimation with a bandwidth of 0.2 µm, the vertical red line represents the maximum of the distribution, and black vertical bars on the x-axis represent individual data points.

d. Similar to c, but for nc14.

e. Distribution of the doc-TAD volumes, as measured by its radius of gyration, shown as violin plots. Distribution for nc11/12 in orange (mean Rg=0.30 µm), nc14 in cyan (mean Rg=0.28 µm). Markers indicate the mean and extreme values of the distribution. The *p*-value was calculated by a two-sided Welch's *t*-test.

f. Effect of reducing the number of barcodes for the UMAP embedding. Data taken from [38]. The UMAP on the left was obtained from 20 barcodes centered around the strong TAD border (see black arrows in the ensemble PWD map on the right). The UMAP on the right was obtained from 20 barcodes centered around a weak TAD border.

g. Segregation of nc11/12 and nc14 nuclei is stable for different UMAP hyperparameters. Plots show UMAPs for different numbers of neighboring sample points (“n_neigh”) and different values for the minimum distance between embedded points (“min_dist”).

Source data for panels b and e are provided as a Source Data file.

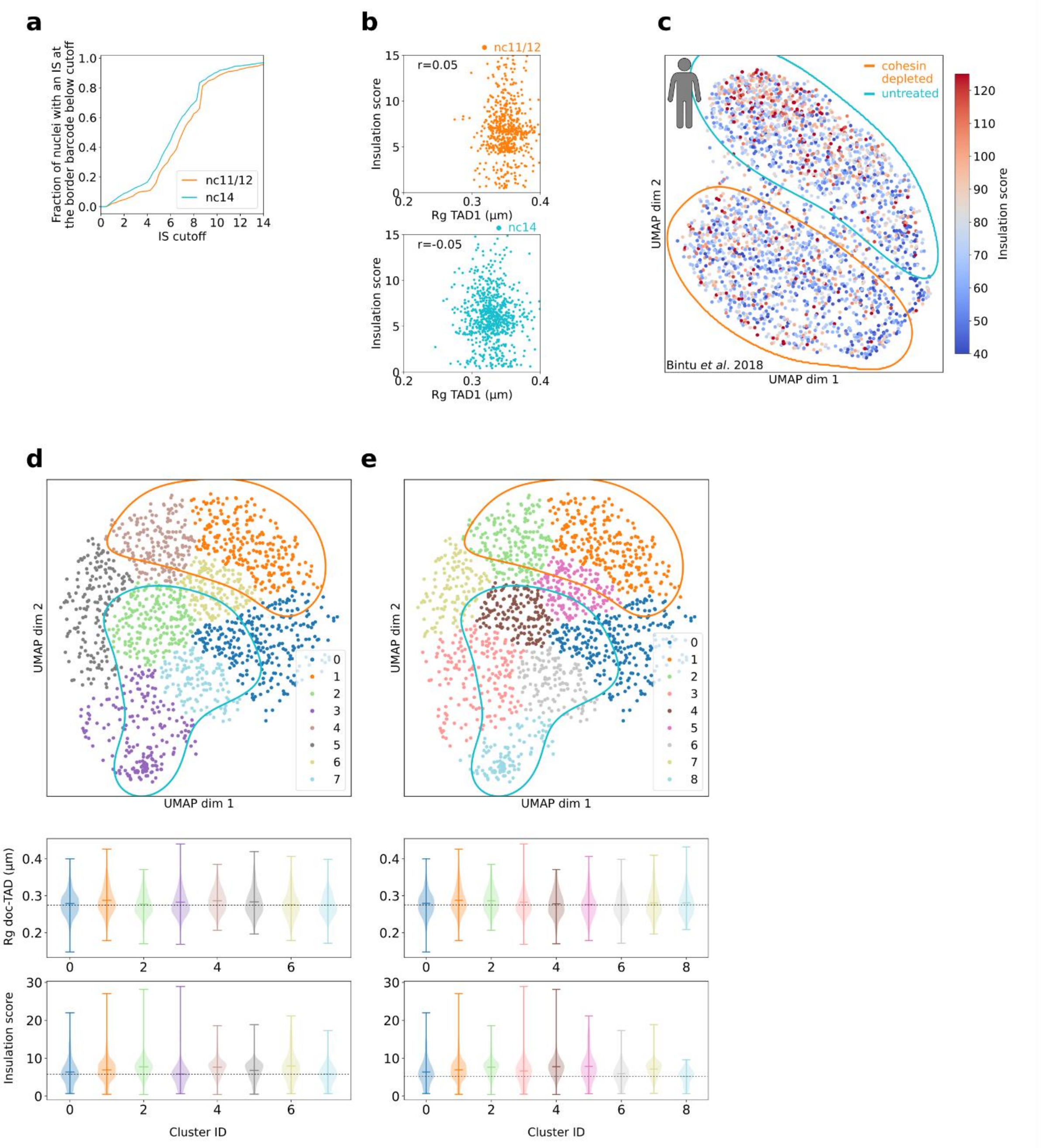

Supplementary Figure 2. TAD insulation is highly heterogeneous at the single nucleus level.

a. Cumulative distribution of the single nucleus IS for nc11/12 (orange) and nc14 (cyan) embryos.

b. Top: scatter plot of insulation score at the border barcode versus radius of gyration of TAD1 in nc11/12. Each point corresponds to a single nucleus. *r* is the Pearson correlation coefficient. Bottom: similar to the top, but for nc14.

c. Same UMAP as in Fig. 1g (data taken from [38]), color-coded by the insulation score at the border barcode (indicated as "strong" border in Fig. S1f).

d-e. Leiden decomposition of UMAP and mappings of the radius of gyration and the insulation scores for each of the Leiden clusters. Leiden clusters are shown in different colors. The only difference between the Leiden clustering of Figs. 2g, S2d and S2e was the resolution used: 0.25 (Fig. 2g), 0.33 (Fig. S2d), and 0.5 (Fig. S2e). This change in

resolution results in 6, 8 and 9 Leiden clusters, respectively. The remaining parameters were kept constant and are reported in the Methods section. Number of nuclei for clusters 0-7 in panel d: 280, 246, 225, 199, 194, 174, 163, and 124, respectively. Number of nuclei for clusters 0-8 in panel e: 272, 250, 194, 186, 169, 167, 128, 127, and 112, respectively. Bottom panels: Markers indicate the mean and extreme values of the distribution.

Source data for all panels are provided as a Source Data file.

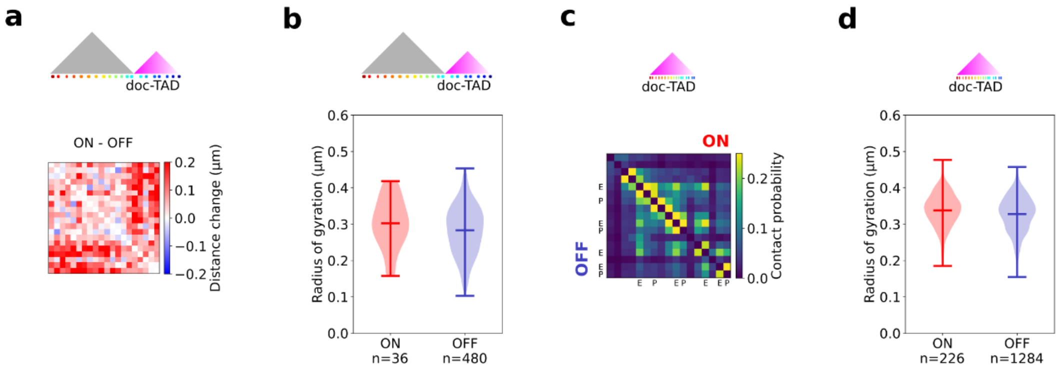

Supplementary Figure 3. Transcriptional activation has a minor impact on TAD volumes but not proximity frequencies.

a. Change in the ePWD map between transcriptionally active ("ON") and inactive ("OFF") nuclei for the Hi-M library that covers TAD1 and the doc-TAD. Distances that are larger in ON nuclei are in red.

b. Violin port of the TAD volume (as measured by the radius of gyration) for the doc-TAD in transcriptionally active ("ON") and inactive ("OFF") nuclei. Markers indicate the mean and extreme values of the distribution. The radius of gyration is 0.30±0.07µm for active and 0.28±0.07µm for inactive nuclei (mean±standard deviation of the distribution).

c. Contact probability map for the high-resolution Hi-M library covering the doc-TAD. Upper right half of the matrix displays the map for active nuclei, and the lower left half for inactive nuclei. Barcodes with cis-regulatory elements (enhancer E, promoter P) are indicated.

d. Similar to panel b, but for the high-resolution Hi-M library. The radius of gyration is 0.34±0.05µm for active and 0.33±0.04µm for inactive nuclei (mean±standard deviation of the distribution).

Source data for all panels are provided as a Source Data file.

a

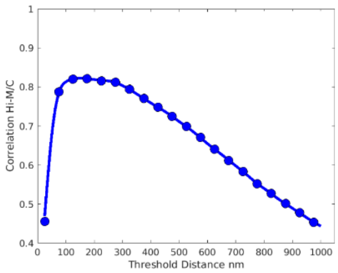

Supplementary Figure 4. Selection of proximity threshold.

a. Cross-correlation between the Hi-C matrix of nc14 embryos [24] and Hi-M matrices for difference proximity thresholds ($d_{threshold}$). Proximity thresholds between 100-300 nm resulted in very similar correlation scores, with $d_{threshold}$ = 250 nm providing higher statistics than lower thresholds.

Supplementary Tables

Supplementary Table 1. List of publicly available data used in this study.

**Dataset**	**Publication**	**GSE/Ref**
ATAC-seq	Hannon et al 2017	GSE86966[https://www.ncbi.nlm.nih.gov/geo/query/acc.cgi?acc=GSE86966]
Chip-seq for RNA pol II	Sun et al 2015	GSE65441[https://www.ncbi.nlm.nih.gov/geo/query/acc.cgi?acc=GSE65441]
Hi-C staged embryos	Ogiyama et al 2018	GSE103625[https://www.ncbi.nlm.nih.gov/geo/query/acc.cgi?acc=GSE103625]

Supplementary Table 2. RNA probes information.

<table>
<tr><th>Probe</th><th>Vector backbone</th><th>Restriction enzyme</th><th>RNA polymerase</th></tr>
<tr><td>Doc1</td><td>pCR II</td><td>EcoRV</td><td>SP6</td></tr>
<tr><td colspan="4">Primer information</td></tr>
<tr><td colspan="2">Primer name</td><td colspan="2">Primer Sequence</td></tr>
<tr><td colspan="2">Doc1_intronic_Forward</td><td colspan="2">CCTATCTCATACGAGTACGAG</td></tr>
<tr><td colspan="2">Doc1_intronic_Reverse</td><td colspan="2">AGGACTTGATTAAGTGGCCTGCC</td></tr>
</table>

Supplementary Table 3. List of genomic positions of the barcodes used in this study.

**Low-Res *doc*-TAD (barcodes: chromosome 3L; fiducial: chromosome 2L)**						
Number	Readout	Coordinate (dm3)		Center	Size (bp)	Number of oligos
		Start	End			
1	RT1	8882600	8886000	8884300	3400	45
2	RT2	8901400	8904000	8902700	2600	45
3	RT3	8918000	8922000	8920000	4000	45
4	RT22	8945000	8949000	8947000	4000	45
5	RT23	8959500	8963000	8961250	3500	45
6	RT24	8972700	8976500	8974600	3800	45
7	RT7	8989000	8992600	8990800	3600	45
8	RT8	9005500	9009100	9007300	3600	45
9	RT9	9020600	9024000	9022300	3400	45
10	RT10	9034500	9039000	9036750	4500	45
11	RT11	8891000	8894500	8892750	3500	45
12	RT12	8909400	8913200	8911300	3800	45
13	RT13	8927000	8931000	8929000	4000	45
14	RT14	8936000	8939800	8937900	3800	45

15	RT15	8952200	8955500	8953850	3300	45
16	RT16	8966500	8969500	8968000	3000	45
17	RT17	8978000	8981700	8979850	3700	45
18	RT18	8995500	8999200	8997350	3700	45
19	RT19	9011000	9014500	9012750	3500	45
20	RT20	9027500	9031000	9029250	3500	45
21	RT_fiducial	9990000	10010000	10000000	20000	267

**High-Res *doc*-TAD**						
Number	Readout	Coordinate (dm3)		Center	Size (bp)	Number of oligos
		Start	End			
1	RT25	8974562	8977955	8976258	3393	50
2	RT26	8977958	8981958	8979958	4000	50
3	RT27	8982073	8985954	8984013	3881	50
4	RT28	8985957	8988814	8987385	2857	50
5	RT29	8988874	8992812	8990843	3938	50
6	RT30	8992815	8995836	8994325	3021	50

7	RT31	8995839	8999945	8997892	4106	50
8	RT32	9000067	9003457	9001762	3390	50
9	RT33	9003460	9006983	9005221	3523	50
10	RT34	9007073	9010836	9008954	3763	50
11	RT35	9010839	9014616	9012727	3777	50
12	RT36	9014655	9018437	9016546	3782	50
13	RT37	9018440	9022234	9020337	3794	50
14	RT38	9022243	9025656	9023949	3413	50
15	RT39	9025809	9030194	9028001	4385	50
16	RT40	9030253	9032999	9031626	2746	50
17	RT41	9033037	9038920	9035978	5883	50
18	RT_fiducial	7882692	7909948	7896320	27256	352

Supplementary Table 4. List of primers for library amplification used in this study.

Low-Res library	
Name	Sequence (5’ >>> 3’)
BB297 (Primer forward)	GACTGGTACTCGCGTGACTTG
BB299 (Primer reverse)	GTAGGGACACCTCTGGACTGG
T7+BB299 (T7 promoter + Primer reverse)	TAATACGACTCACTATAGGGTGTAGGGACACCTCTGGACTGG
BB287-FWD (Primer forward_fiducial)	CGCTCGGTCTCCGTTCGTCTC
BB288-REV (Primer reverse_fiducial)	GGGCTAGGTACAGGGTTCAGC
T7+BB288 (T7 promoter + Primer reverse_fiducial)	TAATACGACTCACTATAGGGTGGGCTAGGTACAGGGTTCAGC

High-Res libraries	
Name	Sequence (5’ >>> 3’)
BB193 (Primer forward)	TTGATCTCGCTGGATCGTTCTGCAATG
BB280 (Primer reverse)	GGGAGTAGGGTCCTTTGTGTG
T7+BB280 (T7 promoter + Primer reverse)	TAATACGACTCACTATAGGGTGGGAGTAGGGTCCTTTGTGTG
