# Supplementary material for: Multiple parameters shape the 3D chromatin structure of single nuclei at the doc locus in Drosophila
Source: Nat Commun. 2022 Sep 14;13:5375. doi: 10.1038/s41467-022-32973-y (PMC9474875; doi:10.1038/s41467-022-32973-y)
Supplement: Supplementary file 3 — Description of additional Supplementary File [file 41467_2022_32973_MOESM3_ESM.pdf]

<u>Descriptions of additional supplementary files</u>

Supplementary Data 1

List of sequences of imaging (io) and adapter oligos used in this study. The adapter oligonucleotides were used to couple the primary HiM probes (see Supplementary Data 2) to the imaging oligonucleotides that are fluorescently labeled at their 3’ end.

Supplementary Data 2

List of sequences of primary Hi-M probes. Each oligonucleotide comprises 5 regions: a forward priming region, a readout region, a genome homology region, a readout region, and a reverse priming region.
